# Typicality and trajectories of problematic and positive behaviors over adolescence in eight countries

**DOI:** 10.3389/fpsyg.2022.991727

**Published:** 2023-01-26

**Authors:** Christy M. Buchanan, Susannah Zietz, Jennifer E. Lansford, Ann T. Skinner, Laura Di Giunta, Kenneth A. Dodge, Sevtap Gurdal, Qin Liu, Qian Long, Paul Oburu, Concetta Pastorelli, Emma Sorbring, Laurence Steinberg, Sombat Tapanya, Liliana Maria Uribe Tirado, Saengduean Yotanyamaneewong, Liane P. Alampay, Suha Al-Hassan, Dario Bacchini, Marc H. Bornstein, Lei Chang, Kirby Deater-Deckard

**Affiliations:** ^1^Department of Psychology, Wake Forest University, Winston-Salem, NC, United States; ^2^Center for Child and Family Policy, Duke University, Durham, NC, United States; ^3^Department of Psychology, Università di Roma “La Sapienza”, Rome, Italy; ^4^Centre for Child and Youth Studies, University West, Trollhättan, Sweden; ^5^Department of Maternal and Child Health and Adolescent Health, Chongqing Medical University, Chongqing, China; ^6^Global Health, Duke Kunshan University, Kunshan, Jiangsu, China; ^7^Department of Educational Psychology, Maseno University, Maseno, Kenya; ^8^Department of Psychology, Temple University, Philadelphia, PA, United States; ^9^Department of Psychology, King Abdulaziz University, Jeddah, Saudi Arabia; ^10^Department of Psychiatry, Chiang Mai University, Chiang Mai, Thailand; ^11^Department of Psychology, University of San Buenaventura, Medellín, Colombia; ^12^Department of Psychology, Ateneo de Manila University, Quezon City, Philippines; ^13^Special Education, Hashemite University, Zarqa, Jordan; ^14^Department of Humanistic Studies, University of Naples “Federico II”, Naples, Italy; ^15^Eunice Kennedy Shriver National Institute of Child Health and Human Development (NIH), Bethesda, MD, United States; ^16^United Nations Children’s Fund (UNICEF), New York, NY, United States; ^17^Institute for Fiscal Studies, London, United Kingdom; ^18^Department of Psychology, University of Macau, Taipa, China; ^19^Psychological and Brain Sciences, University of Massachusetts Amherst, Amherst, MA, United States

**Keywords:** adolescent behavior, externalizing and internalizing behavior, wellbeing, storm and stress, cultures

## Abstract

In this study, we examine the predictions of a storm and stress characterization of adolescence concerning typicality and trajectories of internalizing, externalizing, and wellbeing from late childhood through late adolescence. Using data from the Parenting Across Cultures study, levels and trajectories of these characteristics were analyzed for 1,211 adolescents from 11 cultural groups across eight countries. Data were longitudinal, collected at seven timepoints from 8 to 17 years of age. Results provide more support for a storm and stress characterization with respect to the developmental *trajectories* of behavior and characteristics from childhood to adolescence or across the adolescent years than with respect to *typicality* of behavior. Overall, adolescents’ behavior was more positive than negative in all cultural groups across childhood and adolescence. There was cultural variability in both prevalence and trajectories of behavior. The data provide support for arguments that a more positive and nuanced characterization of adolescence is appropriate and important.

## Introduction

A google search for “stereotypes of teens” quickly reveals a variety of websites addressing negative stereotypes of teenagers. The stereotypes cited range from “hormonal” and “moody” to “irresponsible,” “selfish,” “mean,” and “rebellious” (e.g., Hunt, 2020; The Children’s Society, 2021; Pool, 2022). As the titles of the online articles imply (*8 Stereotypes of Teenagers that We Need to Get Rid Of; Dangers of Teenage Stereotypes; How Stereotypes of Teens Harm Families*), their aim is often to dispel the negative narrative, illuminating the detrimental impact such assumptions can have for teenagers and their families. Such stereotypes are prevalent in U.S. cultural models of adolescence (Busso et al., 2018). The stereotypes have roots in psychological theory dominated by a Western perspective that promoted a storm and stress characterization of adolescence (Hall, 1904; Arnett, 1999). Furthermore, despite questions and concerns raised about this characterization over at least the past half century (e.g., Offer and Schonert-Reichl, 1992), it has been perpetuated by misinterpretations of research on brain development (e.g., National Academies of Sciences, Engineering, and Medicine [NASEM], 2019) and more than a century of research on adolescent behavior that, itself influenced by theory and public health concerns, has focused largely on more negative or problematic aspects of adolescent behavior (e.g., Nichols and Good, 2004; Institute of Medicine and National Research Council, 2011; Hollenstein and Lougheed, 2013). Consistent evidence for *increases* in challenging behaviors such as risk-taking, moodiness, depression, and parent-child conflict, as children move from childhood into and through adolescence, at least in Western contexts, has helped to perpetuate negative stereotypes (Arnett, 1999; Buchanan and Bruton, 2016). Through an illogical leap, characterizations of adolescence often imply that the difficulties are normative, if not universal, and inevitable (Nichols and Good, 2004; Hollenstein and Lougheed, 2013). In other words, the focus on *increases* in certain difficulties relative to childhood can lead to a failure to consider the absolute prevalence, or typicality, of difficult behaviors even at their developmental peak (Hollenstein and Lougheed, 2013; Buchanan and Bruton, 2016).

Across most societies examined there is an adolescent stage that begins with puberty (Dasen, 2000). There is variation in the timing of pubertal changes, but on average they begin around 10–11 years of age. The end of adolescence is more variable across cultures, depending on the timing of transition into adult roles (Dasen). In Western contexts and many non-Western urban contexts, one common marker of the transition out of adolescence is the end of formal schooling (e.g., high school, approximately age 18; National Academies of Sciences, Engineering, and Medicine [NASEM], 2019). In cultures where the transition into adult roles has been further extended, the years from 19 to 25 have sometimes been considered an extension of adolescence, but in recent decades have been more often conceptualized as emerging adulthood (Arnett, 2000). The focus of this paper is limited to the earlier portion, as we examine development from 8 (pre-adolescence) through 17 years of age. Western scholars have identified phases within this overall period of adolescence. Although age ranges for these phases are identified somewhat differently in different sources, they are often identified as early adolescence (beginning as early as 10 years and extending through 12–13 years), middle adolescence (roughly 13 or 14–16 years), and late (16 or 17–18 years) adolescence (e.g., National Academies of Sciences, Engineering, and Medicine [NASEM], 2019; note that the *Journal of Early Adolescence* publishes studies on youth aged 10–14 years). In Western settings, phases are marked by differences in pubertal development, school transitions, and levels of autonomy.

Thus, adolescence often entails much change. In the USA and other developed countries, there are well-documented changes in the body and the brain (Casey et al., 2008), in school settings and expectations (Eccles and Roeser, 2003; Benner, 2011), and in time spent with family, same-sex and oppositive-sex peers, and other activities (Larson et al., 1996; Larson, 2001). Cognitive abilities also change significantly, with the emergence of the capacity for more abstract thought and systematic problem-solving (Byrnes, 2006). Given the number of physical, cognitive, and social changes associated with transitions in and through adolescence, along with the increases in autonomy that occur (National Academies of Sciences, Engineering, and Medicine [NASEM], 2019), it is not surprising that certain difficulties and challenges increase in adolescence compared to childhood in Western contexts and those subject to Western influences (e.g., Qu et al., 2020). The *increase* in difficulties, and the public health challenges created by them, are important to acknowledge and address (e.g., Lee et al., 2014). However, an accurate developmental characterization of adolescence must simultaneously account for the *typicality* of such difficulties (Hollenstein and Lougheed, 2013). Typicality can be defined as the prevalence of a specific problem among adolescents (e.g., the percent of adolescents who exhibit a problem such as binge drinking one or more times in a 2-week period), or the average “level” or frequency of a problem (e.g., the intensity or frequency of unexplained stomach aches). Due to cognitive, biological, and social changes, adolescents might grow more likely than younger children to take risks or more likely to act in ways that diverge from or defy parental values and expectations (Romer et al., 2017). However, knowing whether that increase in the population results in an objectively high prevalence of risk-taking or disobeying parents, in such behaviors becoming normative, provides important context to the developmental increase (Institute of Medicine and National Research Council, 2011; Buchanan and Bruton, 2016). Similarly, the multiple changes of adolescence might produce increases in mood swings or negative mood compared to childhood, but increases alone do not provide a full picture of the typical adolescent; it is also important to know just how common in the population internalizing typically is, and whether the typical adolescent’s mood is characterized by sadness or depression (vs. happiness).

Furthermore, an accurate characterization of adolescence must attend to and incorporate developmental changes and typicality of positive, as well as negative, behaviors. Adolescents might take more risks than children, but also grow in empathy that leads them to take more risks on behalf of others. Their ability to question the *status quo* might lead to more questioning or defiance of authority, but they might also grow more capable of perspective-taking and future-orientation that allows for compromise, conflict resolution, and self-regulation. Scholarship in comparative psychology, ethnography, and anthropology has long questioned the storm and stress characterization of adolescence (e.g., Schlegel and Barry, 1991) and recognized positive developments (Dasen, 2000). More recently, psychologists from the West have also begun to seriously examine the development of positive characteristics, and to incorporate the potential for positive development into developmental theories (Lerner et al., 2005; Wray-Lake et al., 2016; Shubert et al., 2019; Abrams, 2022; Defoe and Romer, 2022), but this approach to conceptualizing adolescent development is still fairly young, and arguably overshadowed by negative stereotypes, expectations, and concerns.

Finally, an accurate characterization of adolescence must account for the impact of culture, consistent with bioecological models of human development (Bronfenbrenner and Morris, 2006). As indicated above, alternative characterizations of adolescence have been uncovered in ethnographic studies of non-Western cultural groups (e.g., Schlegel and Barry, 1991; Dasen, 2000). Cultural differences in values and beliefs (e.g., respect for parental authority; Alampay, 2014; Smetana and Rote, 2019), in experiences leading to adulthood (e.g., amount of time spent in leisure vs. labor; timeline for taking on adult obligations; Dasen, 2000; Larson, 2001), and in stereotypes about adolescent behavior (e.g., Qu et al., 2016, 2020) are among the contextual reasons for different adolescent outcomes. Systematic comparisons of typicality and trajectories of behavior can provide a valuable contribution to knowledge about how best to characterize adolescence as a stage of development.

Developing an accurate characterization of adolescence is important for applied, as well as theoretical, reasons. Parents, teachers, policy-makers, researchers, and others who serve or interact with adolescents can be influenced by the characterizations of adolescence that are rooted in negative cultural stereotypes, which are themselves at least partly rooted in scientific characterizations (Nichols and Good, 2004; Jewell et al., 2019). By explicitly accounting for the typicality of both negative and positive characteristics, alongside developmental changes in both negative and positive characteristics, it is possible to provide a more nuanced and accurate characterization of adolescence, a characterization that can potentially provide a needed corrective to the negative stereotypes that drive much current thinking about and interaction with adolescents, at least in Western contexts (Nichols and Good, 2004; Buchanan and Bruton, 2016). Ethological studies suggest that taking a global approach to understanding adolescence can also contribute to a corrective, by indicating changes that are more universal and possibly inevitable, but also the possibilities for different trajectories of development based on context (Dasen, 2000).

In this paper, we draw on data from the Parenting Across Cultures (PAC) study to examine the typicality of both problematic and positive behaviors and characteristics beginning in late childhood and continuing across the adolescent years. These longitudinal data were gathered from individuals in 11 cultural groups across eight countries seven times from 8 to 17 years of age. Thus, the data provide insight into developmental trajectories as well as typicality of behaviors across several countries. Specifically, we address whether internalizing (i.e., mood and emotional problems including depression and anxiety), externalizing (i.e., problem behaviors including school misconduct, substance use, and aggression), and wellbeing across this decade of life demonstrate the age changes and typicality predicted by a storm and stress characterization.

### Internalizing, externalizing, and wellbeing: Trajectories and typicality

Internalizing and externalizing are among the domains of predicted storm and stress (Arnett, 1999), and because of their importance to public health, are among the most commonly studied aspects of adolescent behavior. Much data support increases in both types of behavior over the adolescent years among teenagers in the U.S. (Substance Abuse and Mental Health Services Administration, 2021; Miech et al., 2022). However, ethnographic research and the increasing extension of mainstream developmental research beyond WEIRD (white, educated, industrialized, rich, democratic) samples (Thalmayer et al., 2021) indicate that the existence or extent of those increases varies across cultures (e.g., Dasen, 2000; U.S. Department of Health and Human Services and Health Resources and Services Administration, 2003; Duell et al., 2016; Rothenberg et al., 2020). Furthermore, a close look at the prevalence of extensive internalizing and externalizing problems shows that they are characteristic of a minority of U.S. adolescents. For example, in 2019, one-fifth of 15–17-year-olds had experienced a major depressive episode within the past year (Daly, 2021). It is concerning that so many teenagers struggle with depression; it is also true that the majority of teenagers are not depressed and, in fact, experience high levels of positive mood on a daily basis (e.g., Larson et al., 2002; Kenny et al., 2016; Gutman et al., 2017). Although a majority of high school seniors in 2021 reported having consumed alcohol at least once in their lifetimes, only one-quarter of them reported having consumed alcohol in the past 30 days and only 12% reported binge drinking (consuming 5 or more drinks in a row) in the past 2 weeks (The Monitoring the Future Study, 2021a,b,c). Furthermore, for both externalizing and internalizing, there have been significant historical changes, with many aspects of externalizing (including alcohol use) having declined markedly over recent decades (Miech et al., 2022) and internalizing problems having risen (Daly, 2021). Such historical changes also reflect cultural changes and add to an overall picture of storm and stress at adolescence as characteristic of a minority, variable, and context-dependent (rather than normative and inevitable).

Given less attention to positive behaviors at adolescence, less is known about their developmental trajectories and typicality. However, evidence suggests that positive behaviors, characteristics, and relationships are highly prevalent (Gutman et al., 2017). In nationally representative studies (Wozniak et al., 2012), three-quarters of adolescents report that helping others who are in difficulty is very important or essential to their education and career goals, and a similar percentage volunteer in their community at least once or twice a month. Eighty-six percent exercise or play sports at least 2–3 times per week, and 80% name a family member as their most valuable relationship (vs. 12% naming a peer). Levels of empathy and perspective-taking are generally high (i.e., adolescents believe these qualities describe themselves well), and increase—at least in some contexts—in the transition to and across adolescence (Lam et al., 2012; Van der Graaff et al., 2014; Miklikowska et al., 2022). Where civic engagement is encouraged and opportunities exist, adolescents tend to be civically involved (Ballard, 2014; Bandura and Cherry, 2020; Oosterhoff et al., 2021), with the extent of involvement demonstrating historical ups and downs, including a “burst” of civic engagement in U.S. adolescents in the 21st century (National Academies of Sciences, Engineering, and Medicine [NASEM], 2019).

### The current study

The current study builds on earlier reports from the Parenting Across Cultures (PAC) study, which examined age trajectories of internalizing and externalizing from age 8–14 years of age across cultures (Rothenberg et al., 2020). In the current study, in addition to examining internalizing and externalizing through age 17 years, we report age trends for wellbeing from 12 to 17 years. Furthermore, to provide the necessary context for an accurate characterization of adolescence, we interpret the data based not just on the trajectory of change with age but also from the perspective of how typical each category of behavior is at its peak.

Based on existing literature, including previous PAC reports, we hypothesized that age *trajectories* in most contexts would mirror the predictions of storm and stress theory: increases in internalizing and externalizing, and decreases in wellbeing from childhood to adolescence or across the adolescent years. We also predicted that the typicality of internalizing and externalizing would be low, and the typicality of wellbeing would be high; specifically, we predicted that even at their peak, absolute levels of externalizing and internalizing would reflect low objective levels of difficulty (with difficulty not normative), and that even at their nadir, levels of wellbeing would reflect relatively high objective positive functioning (with wellbeing normative). We also predicted that there would be differences in typicality and trajectories between cultural groups, with patterns of normative behavior and developmental trajectories of behavior most consistent with storm and stress characterization in Western cultural groups (e.g., Italy, Sweden, U.S.—especially European American) than in non-Western cultural groups (e.g., Brazil, Jordan, Kenya, Thailand).

## Materials and methods

### Participants

Research participants were part of the Parenting Across Cultures study, a longitudinal study started in 2008 with the recruitment of children (*N* = 1,334; *M*age = 8.28 years, *SD* = 0.64 years in wave 1) and their mothers and fathers from nine countries: China, Colombia, Italy (Naples and Rome), Jordan, Kenya, the Philippines, Sweden, Thailand, and the USA (African American, European American, and Hispanic). Because age 17 data were not collected in the Chinese sample, data from China were not included in the current analyses. Thus, the analytic sample included 1,211 children from 11 cultural groups in eight countries (Columbia, *n* = 108; Italy–Naples, *n* = 100; Italy–Rome, *n* = 109; Jordan, *n* = 114; Kenya, *n* = 100, Philippines, *n* = 120; Thailand, *n* = 120; Sweden, *n* = 129; US-African American, *n* = 102; US-European American, *n* = 110; US-Hispanic, *n* = 99).

Selected in proportion approximating the distribution of the student population in each recruitment site, students from both public and private schools were recruited through letters sent home with them. In each site, families participated in annual interviews after their initial recruitment. Measures to address the present research questions were administered in waves 1–10, when children were ages 8–17, on average. At age 17, 71% of the original sample provided data. Continuing participants did not differ from those who did not provide age 17 data on parent age, parent marital status, and number of children in the household, but did differ on child gender and parental education. The study was approved by Institutional Review Boards at universities in each country.

### Procedure and measures

Study measures were translated and back translated and subjected to a process of cultural adaptation to ensure linguistic and conceptual equivalence of the measures. After parents provided informed consent and children provided assent, interviews were conducted face-to-face, over the telephone, or online (depending on the wave of data collection and families’ preferences). Participants were given modest compensation for their time.

Table 1 provides means and standard deviations, for each variable in each site and Bonferroni-adjusted bivariate statistics comparing the levels for each cultural group to the whole sample.

**TABLE 1:** Descriptive statistics for substantive measures at ages 8–10, 12, 14–15, and 17 by cultural group.

Age	Whole sample			Colombia			Italy–Naples			Italy–Rome			Jordan			Kenya			Philippines		
	**M**	**SD**	**R**	**M**	**SD**	**R**	**M**	**SD**	**R**	**M**	**SD**	**R**	**M**	**SD**	**R**	**M**	**SD**	**R**	**M**	**SD**	**R**
**Internalizing**																					
8	14.99	8.49	0–49	**19**.**26**	9.67	4–49	16.07	7.82	0–41	14.37	8.31	0–45	13.90	7.73	0–32	***9***.***18***	6.40	0–29	**18**.**93**	8.12	3–36
9	13.67	8.53	0–51	**16**.**98**	8.05	2–43	14.23	7.67	2–37	14.31	8.79	1–51	12.83	7.93	0–39	***6***.***94***	5.67	0–37	**18**.**43**	8.38	1–41
10	12.01	7.77	0–45	10.76	6.71	0–30	12.36	6.88	1–28	11.69	7.57	1–36	11.71	7.23	0–31	***7***.***71***	3.36	0–16	**18**.**85**	8.01	2–40
12	13.35	8.63	0–51	14.22	8.65	0–39	13.44	8.45	1–40	13.43	7.90	0–39	13.84	7.80	0–36	13.70	8.86	0–37	**18**.**57**	9.10	4–48
14	14.60	9.56	0–52	**17**.**69**	11.78	2–52	15.85	9.90	0–41	15.14	8.98	1–43	12.22	8.71	0–47	13.92	6.81	2–30	**18**.**29**	8.39	3–47
15	13.90	8.91	0–48	15.84	8.61	2–39	14.62	7.83	1–35	14.49	8.80	0–46	12.55	8.83	0–39	13.61	7.37	0–31	**18**.**40**	9.39	4–48
17	14.84	9.21	0–43	14.87	8.24	2–33	16.73	9.22	**2**–**38**	**17**.**82**	8.94	1–40	12.25	7.21	0–42	10.69	6.15	0–23	**18**.**27**	9.44	1–42
**Externalizing**																					
8	9.39	6.59	0–43	10.34	7.57	0–42	10.63	7.09	2–43	9.27	5.34	0–22	**11**.**88**	6.41	0–30	***6***.***71***	4.63	0–19	10.86	7.37	0–41
9	9.95	7.20	0–57	**11**.**92**	6.84	1–33	10.77	6.66	1–40	10.41	7.25	0–57	**12**.**61**	7.59	0–37	8.48	7.15	0–32	11.63	8.27	1–40
10	9.28	6.57	0–42	**7**.**32**	4.97	0–28	9.33	5.54	2–30	9.29	5.94	1–34	**12**.**05**	7.81	0–40	9.20	5.09	0–30	**12**.**40**	7.96	1–42
12	10.57	7.21	0–41	10.46	6.97	1–31	9.61	5.88	0–25	5.88	6.79	0–31	**13**.**72**	8.55	0–40	***7***.***87***	7.02	0–40	12.41	6.67	0–38
14	11.56	7.42	0–45	13.37	8.57	0–45	11.49	6.29	0–26	12.50	7.06	1–31	**13**.**56**	8.19	0–37	***6***.***83***	4.82	0–21	13.24	6.85	2–34
15	10.67	6.77	0–36	**12**.**90**	6.80	1–36	10.71	6.13	0–25	**12**.**48**	6.41	0–27	12.03	7.26	0–35	6.53	5.14	0–20	**13**.**21**	6.36	2–35
17	10.28	7.25	0–46	**12**.**51**	9.27	0–45	10.88	6.37	0–31	**12**.**93**	6.73	2–33	10.72	7.05	0–32	***6***.***49***	5.00	0–21	**12**.**01**	6.43	1–30
**Wellbeing**																					
12	3.93	0.61	1.85–5	***3***.***75***	0.53	2.5–4.95	3.82	0.57	2.4–5	***3***.***67***	0.60	2.45–4.85	***3***.***76***	0.59	2–5	**4**.**22**	0.70	1.85–5	**4**.**12**	0.50	2.95–5
14	3.87	0.62	1.75–5	3.74	0.73	1.85–5	3.74	0.58	1.75–4.95	***3***.***53***	0.58	1.75–4.7	3.78	0.63	2.25–5	**4**.**43**	0.45	2.8–5	4.06	0.54	2.7–5
15	3.59	0.67	1.7–5	3.74	0.54	2.4–4.75	3.55	0.64	2.15–5	3.44	0.61	1.85–4.7	3.40	0.75	1.95–5	**3**.**93**	0.62	2.2–5	3.73	0.66	2.1–4.95
17	3.66	0.68	1–5	3.85	0.56	2.5–5	3.56	0.63	2.25–4.95	***3***.***39***	0.61	1.75–4.5	***3***.***36***	0.78	1–4.85	**4**.**25**	0.57	2.95–5	3.82	0.57	2.6–5
**Age**	**Sweden**			**Thailand**			**US African American**			**US European American**			**US Hispanic**		
	**M**	**SD**	**R**	**M**	**SD**	**R**	**M**	**SD**	**R**	**M**	**SD**	**R**	**M**	**SD**	**R**
**Internalizing**															
8	13.17	8.29	1–39	13.89	8.11	0–36	15.21	9.05	0–40	14.56	6.89	1–30	15.68	8.33	0–36
9	***10.27***	7.56	0–42	15.26	7.86	0–40	12.55	8.93	0–39	14.30	8.36	0–35	13.22	8.93	0–37
10	***9.12***	6.57	0–30	**14.95**	8.38	0–45	11.03	7.64	0–30	12.21	7.61	0–32	11.01	8.77	0–36
12	***8.96***	6.31	0–31	14.77	8.33	1–35	12.12	8.97	0–42	13.29	9.52	0–51	***9.07***	6.84	0–27
14	***11.25***	8.01	0–34	15.94	9.17	0–40	12.24	10.05	0–48	16.25	11.21	0–52	11.64	8.33	0–32
15	12.51	8.01	0–37	14.31	8.44	0–35	***10.23***	9.51	0–39	14.33	10.04	0–42	11.40	7.95	1–40
17	12.19	7.66	0–30	17.02	9.01	0–41	***11.30***	11.30	0–43	16.47	10.32	0–40	11.74	9.44	0–42
**Externalizing**															
8	8.15	5.16	0–31	9.31	7.10	0–28	9.03	7.77	0–34	8.46	5.16	1–25	8.01	6.21	0–29
9	8.14	6.00	1–47	10.26	7.31	0–33	8.19	7.50	0–36	8.47	5.82	0–28	**7.24**	6.16	0–26
10	**7.06**	4.62	0–25	10.74	6.74	0–30	8.40	6.62	0–29	8.47	5.96	0–33	**6.79**	7.28	0–35
12	9.18	4.95	2–22	11.71	7.99	0–41	10.11	8.65	0–39	10.66	6.22	0–28	8.26	6.45	0–32
14	10.66	5.88	1–32	13.51	7.78	1–35	9.34	7.91	0–33	12.09	7.61	0–40	8.97	6.73	0–34
15	10.89	6.02	1–29	**12.54**	7.39	0–33	**7.07**	6.51	0–27	9.71	6.01	0–25	**7.77**	5.70	0–29
17	8.66	5.85	0–30	11.05	7.47	0–31	**7.25**	7.81	0–46	9.75	7.24	0–39	***6.98***	5.78	0–21
**Wellbeing**															
12	**4.14**	0.43	2.6–5	***3.59***	0.62	1.95–5	4.15	0.61	2.45–5	4.01	0.49	2.35–5	**4.12**	0.60	2.15–5
14	3.88	0.58	2.4–4.85	3.72	0.55	2.2–4.95	3.99	0.65	2.4–5	3.87	0.53	2.4–5	4.02	0.53	2.7–4.9
15	3.49	0.64	2.2–5	3.53	0.59	2–4.9	3.63	0.75	1.7–5	3.53	0.68	1.85–4.9	3.68	0.69	2.2–4.95
17	3.64	0.55	2.3–4.8	3.77	0.65	2.05–5	***3.71***	0.74	1.8–5	3.43	0.74	1.75–4.75	3.88	0.63	2.6–5

Significantly higher or lower internalizing, externalizing, and wellbeing compared to the overall sample mean are bolded or italicized, respectively.

#### Demographics

Child gender and number of years of mother and father education at the beginning of the study were included in the analyses as covariates.

#### Internalizing and externalizing behavior

Youth completed the Youth Self-Report Form of the Child Behavior Checklist (Achenbach et al., 2001) at ages 8–10, 12, 14–15, and 17; they rated how true each item was during the last 6 months (0 = not true, 1 = somewhat or sometimes true, and 2 = very or often true). The Internalizing Behavior scale summed across 29 items and measured behaviors and emotions such as loneliness, self-consciousness, nervousness, sadness, and anxiety (possible range 0–58). The Externalizing Behavior scale summed across 30 items and captured behaviors such as lying, truancy, vandalism, bullying, disobedience, and physical violence (possible range 0–60). The Achenbach measures are widely used in international research, with translations in over 100 languages and strong, well-documented psychometric properties (e.g., Achenbach et al., 2001). Although the Youth Self-Report was originally designed to be completed by children aged 11–18 whose reading level is advanced enough to complete the measure alone, the items are comparable to items in parallel parent and teacher report versions of the measure appropriate for children as young as 6. Trained interviewers administered the measure orally in the initial years and recorded the children’s responses to avoid the concern about whether children would be able to read the items. Previous research also has demonstrated that children ages 7–10 years are able to make valid reports on the YSR (e.g., Ebesutani et al., 2011). Both the externalizing and internalizing subscales demonstrated strong reliability over time (externalizing α = 0.84; internalizing α = 0.83) and across cultural groups (externalizing α = 0.85; internalizing α = 0.83) in the present sample and have been successfully used in these cultural groups in prior work (e.g., Rescorla et al., 2007; Deater-Deckard et al., 2018; Lansford et al., 2018). Higher scores indicated greater externalizing/internalizing problems.

#### Adolescent wellbeing

Youth self-reported on their wellbeing at ages 12, 14, 15, and 17 using the EPOCH measure of adolescent wellbeing (Kern et al., 2016). The EPOCH measures five different positive youth characteristics comprising adolescent wellbeing and thriving (Kern et al., 2016). These five characteristics are Engagement (being absorbed and involved in an activity or the world itself), Perseverance (the tenacity to stick with things and pursue a goal despite challenges), Optimism (having a sense of hope and confidence about the future), Connectedness (feeling loved, supported, and valued by others), and Happiness (a general feeling of cheer and contentment with life). Each of the five characteristics is assessed using four items rated on a 1 = not at all like me to 5 = very much like me scale. To ensure that the adolescent wellbeing scale was suitable for use in our sample, we examined measurement invariance across cultural groups using the alignment method (Asparouhov and Muthén, 2014). Asparouhov and Muthén (2014) suggest that approximate measurement invariance is attained if less than 20–25% of parameters register measures. Overall, level of non-invariance for child wellbeing at ages 12 (5.97%), 14 (7.95%), 15 (5.11%), and 17 (4.26%) fell below the 25% threshold indicating acceptable measurement invariance across groups. In addition, we conducted a confirmatory factor analysis of the EPOCH subscales at each age they were collected. We found that the best fitting model for each wave was a higher-order factor model with each of the EPOCH constructs loading on one adolescent wellbeing factor. Because the best fitting model was a higher-order factor model and not an overall factor with all items, we then extracted factor scores of adolescent wellbeing for each participant at ages 12, 14, 15, and 17 to be used in the analysis. For ease of interpretation, descriptive statistics for an average sum score are found in Table 1.

### Analysis plan

We estimated a series of latent growth curve (LGC) models. To begin, we estimated a series of single-group unconditional LGC models in each cultural group separately to examine the nature of (1) internalizing behavior from ages 8–17, (2) externalizing behavior from ages 8–17, and (3) wellbeing from ages 12–17. Using a maximum likelihood robust (MLR) estimator in Mplus to adjust for non-normality in the outcomes, in each group, we compared three different functional forms of growth to determine which best captured changes in wellbeing over time and six different functional forms to determine which best captured changes in internalizing and externalizing over time. These included: (1) an intercept-only LGC model that allowed adolescents to vary in internalizing and externalizing behavior at age 8 and wellbeing at age 12 (intercept), but not vary in rate of change in these constructs over time (slope); (2) linear LGC models with heteroskedastic residuals in each cultural group where adolescents were able to vary in their intercept and slope, and slope was assumed to be constant (linear) over time; (3) a quadratic LGC model where adolescents were allowed to vary in their intercept and slope, and a quadratic term was estimated allowing slope to accelerate or decelerate over time. For the internalizing and externalizing models only, a set of three piecewise linear LGC models were estimated, (4) with a knot point of age 10, (5) with a knot point of age 12, (6) and a final one with a knot point of age 14. In these models, two different linear slopes were estimated: one capturing rate of change in the construct before the knot point and one capturing rate of change in the construct after the knot point. For instance, for a knot point of age 10, one slope was calculated between ages 8 and 10, and one between ages 12 and 17. These latter analyses were conducted to examine the possibility of changes in trajectories associated specifically with transitions into adolescence, or from early to middle adolescence.

Following convention (Bollen and Curran, 2006), for each cultural group, we tested model fit among nested models with Chi-square likelihood ratio tests using the Satorra–Bentler scaled Chi-square for MLR estimators, and the best fitting model was retained. Since the piecewise linear models were not nested in the linear or quadratic, the fit of the quadratic and piecewise linear models was compared using the Akaike Information Criterion (AIC) and Bayesian Information Criterion (BIC) indices. The model with the lower AIC/BIC values indicated better fit to the data. Additionally, the fit of all LGCs was evaluated according to the recommended fit index cutoff values of RMSEA < 0.08 and CFI/TLI > 0.95 (MacCallum et al., 1996). Once our final group-specific unconditional LCG models were estimated, we added the demographic predictors of child gender and mother and father years of education.

## Results

### Descriptive statistics

Means and standard deviations of adolescent internalizing, externalizing, and wellbeing can be found in Table 1. In what follows, we summarize data with respect to typicality of internalizing, externalizing, and wellbeing. In other words, we focus on the absolute levels of each, and their implications for the extent to which adolescents report experiencing each outcome even at their worst (i.e., highest internalizing or externalizing, lowest wellbeing).

#### Typicality of internalizing

##### Typicality of internalizing across the whole sample

The possible range of internalizing scores was zero to 58. Across the whole sample, the average absolute value of internalizing was highest at age 8 (*M* = 14.99, range 0–49), prior to adolescence. Given that this average represents the sum of scores across 29 items, this mean is equivalent to an score of 0.52 on the original scale, a response that is between “not true” and “somewhat or sometimes true.” At age 17, in late adolescence, the average absolute value of internalizing for the whole sample was similar to that at age 8 (*M* = 14.84, range = 0–43), equivalent to a score of 0.51 on the original scale.

##### Typicality of internalizing by cultural group

For most cultural groups, at most ages, levels of adolescent internalizing were not significantly different from the average levels of internalizing across all sites. However, there were significantly *higher* levels of adolescent internalizing at all timepoints in the Philippines; at ages 8, 9, and 14 in Colombia; at age 17 in Rome; and at age 10 in Thailand. Compared to the average levels of internalizing across all groups, there were significantly *lower* levels of internalizing at ages 8, 9, and 10 in Kenya; at ages 9, 10, 12, and 14 in Sweden; at ages 15 and 17 in the U.S. African American sample; and at age 12 in the US Hispanic sample.

Internalizing peaked at age 8 in Colombia (*M* = 19.26), the Philippines (*M* = 18.93), the U.S. Hispanic sample (*M* = 15.68), the U.S. African American sample (*M* = 15.21), Jordan (*M* = 13.90), and Sweden (*M* = 13.17); at age 14 in Kenya (*M* = 13.92); and at age 17 in Rome (*M* = 17.82), Thailand (*M* = 17.02), the US European American sample (*M* = 16.47), and in Naples (*M* = 6.73). The lowest peak (Sweden, age 8) is equivalent to a score of 0.45 on the original scale and the highest peak is equivalent to a score of 0.66 (Colombia, age 8). Thus, at its peak, the average internalizing across ages and countries remains between “not at all” and “somewhat or sometimes true” on the original scale. For most of the countries examined, the peak of internalizing occurs in childhood, although for some it occurs in late adolescence.

#### Typicality of externalizing

##### Typicality of externalizing across the whole sample

The possible range of externalizing scores was zero to 60. Across the whole sample, the average absolute value of externalizing was highest at age 14 (*M* = 11.56, *range* = 0–45). Given that this average represents the sum of scores across 30 items, this mean is equivalent to a score of 0.39 on the original scale, a response that is between “not true” and “somewhat or sometimes true.” At age 8, prior to adolescence, the average absolute value of externalizing for the whole sample was 9.39 (*range* = 0–43), equivalent to a score of 0.31 on the original scale. At age 17, in late adolescence, the average absolute value of externalizing for the whole sample was 10.28 (*range* = 0–46), equivalent to a score of 0.34 on the original scale.

##### Typicality of externalizing by cultural group

For most cultural groups, at most ages, levels of adolescent externalizing were not significantly different from the average levels of externalizing across all sites. However, there were significantly *higher* levels of externalizing at age 9, 15, and 17 in Colombia; across ages 8–14 in Jordan; at ages 15 and 17 in Rome; at ages 10, 15, and 17 in the Philippines; and at age 15 in Thailand. Compared to the average levels of externalizing across all groups, there were significantly *lower* levels of externalizing at age 10 in Colombia; age 10 in Sweden; at ages 15 and 17 in the U.S. African American sample; and at ages 9, 10, and 17 in the U.S. Hispanic sample.

Externalizing peaked at age 10 in Kenya (*M* = 9.20); at age 12 in Jordan (*M* = 13.72), and in the U.S. African American sample (*M* = 10.11); at age 14 in Thailand (*M* = 13.51), Colombia (*M* = 13.37), the Philippines (*M* = 13.24), Rome (*M* = 12.50), the U.S. European American sample (*M* = 12.09), Naples (*M* = 11.49), and the U.S. Hispanic sample (*M* = 8.97); and at age 15 in Sweden (*M* = 10.89). The lowest peak (U.S. Hispanic sample, age 14) is equivalent to a score of 0.30 on the original scale and the highest peak is equivalent to a score of 0.46 (Jordan, age 12). Thus, at its peak, externalizing across ages and countries remains between “not at all” and “somewhat or sometimes true” on the original scale. For most of the countries examined, the peak occurs at age 14.

#### Typicality of wellbeing

##### Typicality of wellbeing across the whole sample

The possible range of wellbeing scores was 1–5. Across the whole sample, the average absolute value of wellbeing was lowest at age 15 (*M* = 3.59, *range* = 1.7–5), a score just above the midpoint of the scale (indicating “somewhat more true of me than untrue”). At age 12, in early adolescence, the average absolute value of wellbeing for the whole sample was 3.93 (*range* = 1.85–5), and this was the highest wellbeing score for the whole sample. At age 17, in late adolescence, the average absolute value of wellbeing for the whole sample was 3.66 (*range* = 1–5).

##### Typicality of wellbeing by cultural group

For most cultural groups, at most ages, levels of adolescent wellbeing were not significantly different from the average levels of wellbeing across all sites. However, there were significantly *higher* levels of adolescent wellbeing at all ages in Kenya; and at age 12 in the Philippines, Sweden, and in the U.S. African American sample. Compared to the average levels of wellbeing across all groups, there were significantly *lower* levels of adolescent wellbeing at ages 12, 14, and 17 in Rome; at age 12 in Colombia and Thailand; and at age 17 in the U.S. European American sample.

Mean levels of wellbeing were at their lowest at age 14 and 15 in Colombia (*M* = 3.74 at both times); at age 15 in Rome (*M* = 3.44), Sweden (*M* = 3.49), Thailand (*M* = 3.53), Naples (*M* = 3.55), the U.S. African American sample (*M* = 3.63), the U.S. Hispanic sample (*M* = 3.68), the Philippines (*M* = 3.73), and Kenya (*M* = 3.93); and at age 17 in Jordan (*M* = 3.36), and in the U.S. European American sample (*M* = 3.43). The range of these averages is from 3.36 (Jordan, age 17) to 3.93 (Kenya, age 15). Thus, at its worst, average wellbeing across ages and countries indicates moderate to high wellbeing.

### Trajectories of internalizing, externalizing, and wellbeing across adolescence

**FIGURE 1:**
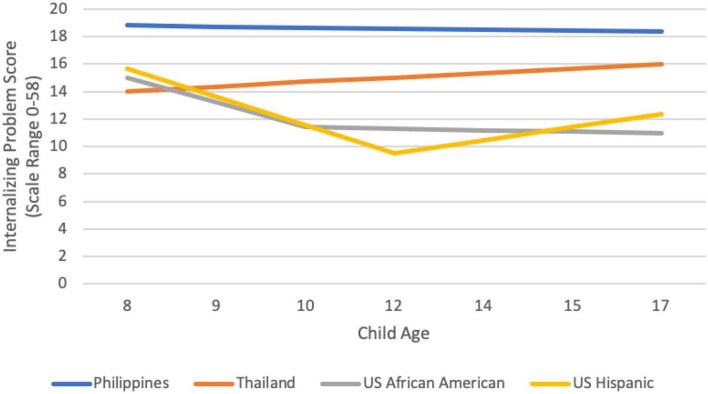
Cultural group-specific observed internalizing trajectories.

**FIGURE 2:**
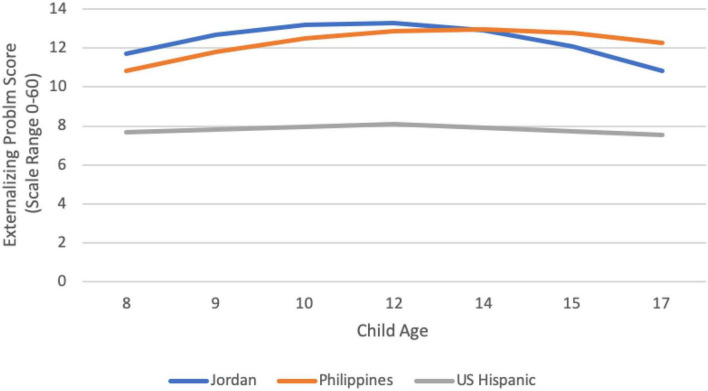
Cultural group-specific observed externalizing trajectories.

**FIGURE 3:**
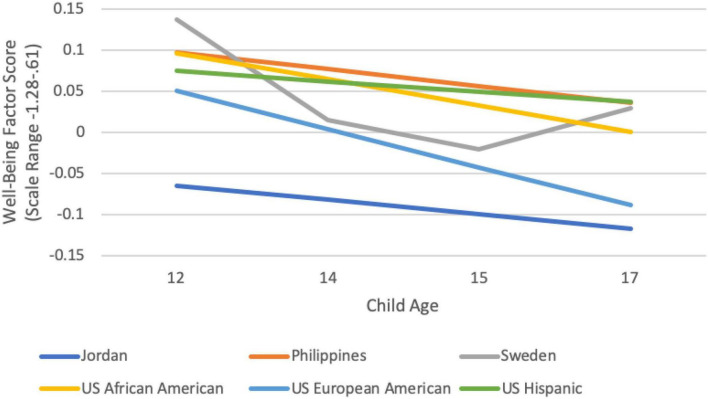
Cultural group-specific observed wellbeing trajectories.

We examined heterogeneity in the trajectory of adolescent externalizing behavior, internalizing behavior, and wellbeing in each cultural group through identifying the optimal functional form of growth that characterized a group’s mean trajectory of these constructs (Curran et al., 2004). Because we did not find the same optimal functional form for each cultural group, we present group-by-group results below. We interpret data only when the final model fit the data well according to omnibus measures of model fit. Group-specific observed internalizing, externalizing, and wellbeing trajectories are depicted in Figures 1–3, respectively. We are able to compare model intercepts across countries and have compared model slopes among countries with the same functional form.

#### Colombia, Italy, and Kenya

No functional form for the Colombia, Italy–Naples, Italy–Rome, or Kenya internalizing, externalizing, and adolescent wellbeing models achieved appropriate levels of model fit. Therefore, we refrain from interpreting these models further.

#### Jordan

No functional form for internalizing achieved appropriate levels of model fit. For child externalizing, a quadratic model best fit the data and fit well (RMSEA = 0.031, 90% CI.000, 0.090; CFI/TLI = 0.988/0.987). The average externalizing score at age 8 was 11.72 (*p* = 0.00) and the initial rate of change between ages 8 and 9 was 1.18 points/year (*p* = 0.01), though this change decreased 0.221 (*p* = 0.00) with each year from ages 9–17. Externalizing declined after peaking at age 12. There was significant variability of the intercept, slope, and quadratic function. Child gender was significantly associated both with the initial slope (β = -1.78, *p* = 0.23) and quadratic rate of change (β = 0.38, *p* = 0.00). The initial positive slope between ages 8 and 9 was significant only for males (β = 2.104, *p* = 0.001) but not females (β = 0.282, *p* = 0.645). Additionally, the decrease in this change from ages 9–17 was significant only for males (β = –0.420, *p* = 0.000) but not females (β = –0.035, *p* = 0.703). With regard to adolescent wellbeing, a linear model was the best fit to the data and fit well (RMSEA = 0.078, 90% CI.000, 0.157; CFI/TLI = 0.925/0.936). The average wellbeing score at age 12 was –0.065 (*p* = 0.043) and did not significantly change over time (β = –0.018, *p* = 0.161). Neither child gender nor parent education were significantly associated with the intercepts or slopes.

#### Philippines

For internalizing, the linear model was the best fit to the data and fit well (RMSEA = 0.047, 90% CI:0.000, 0.093; CFI/TLI = 0.973/0.975). The average internalizing score at age 8 was 18.81 (*p* = 0.00), but there was no significant change over time (β = –0.047, *p* = 0.702). Neither child gender nor parent education was significantly associated with the internalizing intercept or slope. For externalizing, the quadratic model was the best fit to the data and fit well (RMSEA = 0.020, 90% CI:0.000, 0.083; CFI/TLI = 0.995/0.995). The average externalizing score at age 8 was 10.813, the initial rate of change from ages 8–9 was 1.134 (*p* = 0.001), but that rate of change decreased at a rate of 0.148 per year (*p* = 0.011). Externalizing declined after peaking at age 14. Neither child gender nor parent education was significantly associated with the intercepts or slopes. With regard to adolescent wellbeing, a linear model was the best fit to the data and fit well (RMSEA = 0.015, 90% CI.000, 0.140; CFI/TLI = 0.999/0.900). The average wellbeing score at age 12 was 0.097 (*p* = 0.000) and did not significantly change over time (β = 0.002, *p* = 0.062). Child gender and parent education were not significantly associated with the adolescent wellbeing intercept and slope.

#### Sweden

No functional form for internalizing or externalizing achieved appropriate levels of model fit. With regard to adolescent wellbeing, a quadratic model was the best fit to the data and fit well (RMSEA = 0.000, 90% CI.000, 0.190; CFI/TLI = 1.00/1.00). The average wellbeing score at age 12 was 0.137 (*p* = 0.000) and the initial rate of change between ages 8 and 9 was –0.164 points/year, though this change increases by 0.86 with each year. Wellbeing increased after hitting its lowest point at age 15. Neither child gender nor parent education was significantly associated with the intercepts or slopes.

#### Thailand

No functional form for adolescent wellbeing or adolescent externalizing achieved appropriate levels of model fit. With regard to adolescent internalizing, a piecewise model with a knot point at age 10 was the best fit and fit the data well (RMSEA = 0.014 90% CI.000, 0.081: CFI/TLI = 0.997/0.997). The average internalizing score at age 8 was 14.01 (*p* = 0.000), but neither of the two slopes were significant (β ages 8–10:0.0352, *p* = 0.442; β ages 10–17:0.321, *p* = 0.208). However, there were significant variances for the intercepts and both slopes. The intercept was significantly associated with maternal education (β = 0.581, *p* = 0.045), with higher levels of maternal education being associated with higher levels of internalizing at age 8.

#### U.S. African American

No functional form for externalizing ever achieved appropriate levels of model fit. With regard to adolescent internalizing, a piecewise model with a knot point at age 10 was the best fit and fit the data well (RMSEA = 0.061, 90% CI.000, 0.113: CFI/TLI = 0.948/0.942; SRMR = 0.077). At age 8, the average internalizing score was 15.01 (*p* = 0.00), and significantly decreased from age 8–10 each year at a rate of 1.784 (*p* = 0.000). There was no significant change in internalizing from years 12–17 (β = –0.122, *p* = 0.685). However, after controlling for covariates, the slope from ages 8–10 became non-significant. The slope from ages 12–17 remained non-significant overall, but varied significantly by child gender (β = 0.989, *p* = 0.00). There was a significant decrease in internalizing from ages 12–17 for boys (β = −1.047, *p* = 0.006), but no significant change for girls (β = 0.790, *p* = 0.058). With regard to adolescent wellbeing, a linear model was the best fit to the data and fit well (RMSEA = 0.000, 90% CI.000, 0.136; CFI/TLI = 1.00/1.00). The average wellbeing score at age 12 was 0.096 (*p* = 0.003) and significantly decreased each year at a rate of 0.032 points (*p* = 0.041). Neither gender nor parent education was significantly associated with the intercepts or slopes.

#### U.S. European American

No functional form for internalizing or externalizing achieved appropriate levels of model fit. With regard to adolescent wellbeing, a linear model was the best fit to the data and fit well (RMSEA = 0.064, 90% CI.000, 0.164; CFI/TLI = 0.981/0.977). The average wellbeing score at age 12 was not significantly different from 0 (α = 0.050, *p* = 0.067) and significantly decreased each year at a rate of 0.046 points (*p* = 0.000). Neither gender nor parent education was significantly associated with the intercepts or slopes. Father’s education was associated with model slope (β = –0.008, *p* = 0.037). The simple slopes of wellbeing decreased significantly over time only for those who had fathers with medium (β = –0.038, *p* = 0.001) or high (β = –0.105, *p* = 0.002) levels of father education, but not for those who had fathers with low levels of education (β = 0.029, *p* = 39).

#### U.S. Hispanic

With regard to adolescent internalizing, a piecewise model was the best fit to the data and fit well (RMSEA = 0.059, 90% CI.000, 0.112; CFI/TLI = 0.950/0.945). The average internalizing at age 8 was 15.632 (*p* = 0.00), which decreased 2.042 units (*p* = 0.00) per year on average from ages 8–12, but increased 0.96 units per year on average from ages 14–17 (*p* = 0.01). The slope from ages 8–12 was significantly associated with father education (β = 0.34, *p* = 0.00). There was a significant increase in internalizing from ages 8–12 for adolescents whose fathers had a high level of education (β = 3.220, *p* = 0.004) and a significant decrease in internalizing from ages 8–12 for adolescents whose fathers had a low level of education (β = −2.216, *p* = 0.00), while there was no significant decrease in internalizing from ages 8–12 for adolescents whose fathers had the mean level of father education (β = 0.503, *p* = 0.403). With regard to externalizing, a piecewise model with a knot point at age 12 was the best fit and fit the data well (RMSEA = 0.035, 90% CI.000, 0.097: CFI/TLI = 0.984/0.982). At age 8, the average externalizing score was 7.69 (*p* = 0.00), but neither the slope from ages 8–12 (β = 0.135 *p* = 0.59) nor 12–17 (β = –0.179 *p* = 0.550) was significant. However, child gender was significantly associated with the slope from ages 12–17 (β = 1.830, *p* = 0.002). There was a significant decrease in externalizing from ages 12–17 for girls (β = −1.036 *p* = 0.013), but a significant increase in externalizing from ages 12–17 for boys (β = 0.707 *p* = 0.048). With regard to adolescent wellbeing, a linear model was the best fit to the data and fit well (RMSEA = 0.035, 90% CI.000, 0.164; CFI/TLI = 0.993/0.992). The average wellbeing score at age 12 was 0.075 (*p* = 0.029) and did not significantly change over time (β = –0.012, *p* = 0.317). Neither gender nor parent education was associated with the intercepts or slopes.

## Discussion

The current study advances understanding of the accuracy and value of a storm and stress characterization about adolescence. In the U.S. and other Western countries, adolescence is marked by a confluence of physical, cognitive, and social changes (National Academies of Sciences, Engineering, and Medicine [NASEM], 2019) that require adaptation by adolescents, their families, and others who work and interact with them. Adolescence is also a time of transition into adulthood, and thus a time where adolescents desire and typically experience greater autonomy and less adult supervision (National Academies of Sciences, Engineering, and Medicine [NASEM], 2019). As a result of these developments, it has long been recognized that certain difficulties and challenges increase in adolescence compared to childhood in Western contexts and those subject to Western influences (e.g., Arnett, 1999; Qu et al., 2020). Increased difficulties and challenges in the domains of internalizing, externalizing, and wellbeing have contributed to a dominant narrative of storm and stress at adolescence (e.g., Hollenstein and Lougheed, 2013; Buchanan and Bruton, 2016).

Often neglected in the storm and stress narrative is a consideration of *typicality*, or absolute prevalence of such difficulties, even at their peak (Nichols and Good, 2004; Hollenstein and Lougheed, 2013). Certain difficulties might increase compared to childhood, but this does not in itself mean that the problems are so frequent, intense, or common that adolescent behavior is best characterized by these difficulties. In other words, developmental increases alone do not mean a behavior becomes normative. Similarly, there might be positive characteristics that characterize adolescence, but that have been neglected or overlooked in the Western focus on problems (Buchanan and Bruton, 2016; Busso et al., 2018). Longstanding ethnographic and anthropological research point to alternative and more positive characterizations of adolescence, often based on qualitative data from non-Western societies (e.g., Schlegel and Barry, 1991; Dasen, 2000). Cultural differences in values and beliefs (e.g., respect for parental authority; Alampay, 2014; Smetana and Rote, 2019), experiences leading to adulthood (e.g., amount of time spent in leisure vs. labor; timeline for taking on adult obligations; Dasen, 2000; Larson, 2001), and stereotypes about adolescent behavior (e.g., Qu et al., 2016) are among the reasons for different adolescent outcomes. Such cultural differences are also predicted by a bioecological model of human development (Bronfenbrenner and Morris, 2006). In sum, an accurate characterization of adolescence will not only incorporate typicality and positive characteristics (along with age trajectories), but also a cultural perspective. Thus, the current study uses cross-cultural longitudinal data extending from childhood through early, middle, and late adolescence to examine the typicality and trajectories of difficulties (internalizing and externalizing) and positive functioning (wellbeing) across cultural groups.

Our study builds on earlier reports from the PAC study that examined developmental trajectories of internalizing and externalizing longitudinally from age eight to 14 years of age (Rothenberg et al., 2020). In the current report, in addition to extending the study of developmental trends for internalizing and externalizing through age 17 years, we examined developmental trends for wellbeing from age 12–17 years. Furthermore, in order to provide the necessary context for an accurate characterization of adolescence, we interpret the data not just based on the trajectory of change with age but also from the perspective of typicality, meaning how typical the behaviors or characteristics in each category are of adolescents, even after any increase (for negative characteristics) or decline (for positive characteristics). We examine these trends for adolescents in 11 cultural groups across eight countries.

Based on existing literature, including previous PAC reports, we predicted that developmental *trajectories* in most contexts would mirror the predictions of storm and stress theory, showing increases in externalizing and internalizing, and decreases in wellbeing, from childhood to adolescence and across the adolescent years. We also predicted that neither high levels of externalizing and internalizing nor low levels of wellbeing would be normative for adolescents, even at the point in development where negative behaviors and characteristics peaked and where positive characteristics hit bottom. We also predicted cultural variability in typicality and trajectories, such that typical behavior and trajectories of behavior consistent with a storm and stress characterization would be more common in Western cultural groups (e.g., Italy, Sweden, U.S.—especially European American) than in non-Western cultural groups (e.g., Columbia, Jordan, Kenya, Thailand).

### Typicality of internalizing, externalizing, and (lack of) wellbeing across cultural groups

A storm and stress characterization of adolescence predicts an increase in negative behaviors at adolescence, and at least in the initial instantiation (Hall, 1904), claimed that such behaviors were normative and widespread, if not universal. For example, Hall wrote: “…*normal* children often pass through stages of passionate cruelty, laziness, lying and thievery…” (italics added, Vol. I, p. 334–335) and “*All* boys develop a greatly increased propensity to fight at puberty, and although most of them while pretending to give way completely seem very terrible in their rage…” (italics added, Vol. I, p. 356). Modern day stereotypes of adolescence derived from this characterization are not so extreme but imply the same: not simply increases in difficult behavior with age but also widespread, common, normative difficult behavior (e.g., disobedience, negativity) (Nichols and Good, 2004; Buchanan and Bruton, 2016). Websites and books marketed to parents or others who work with adolescents are often framed by this characterization, even if their content is intended to dispel such stereotypes and offer guidance on how to avoid the extreme problems they suggest (e.g., Bradley, 2002; Danesi, 2003; Miller, 2021; McKinney, 2022). Thus, the implication of a storm and stress characterization is that negative characteristics such as externalizing and internalizing are typical during adolescence. Although less is said about positive characteristics, the parallel assumption is that wellbeing is compromised during this time, that low levels of wellbeing are common. The data from our examination of mean levels of externalizing, internalizing, and wellbeing across countries and ages do not support this characterization.^1^

Across cultural groups, the highest average score for internalizing symptoms (e.g., loneliness, sadness, anxiety) for the whole sample occurred among 8-year-olds, not during adolescence, and represented a typical frequency of symptoms between “not true of me” and “somewhat or sometimes true of me” within the past 6 months. This is consistent with other data on typicality of internalizing, which suggest a low average prevalence (e.g., Kenny et al., 2016; Gutman et al., 2017; Daly, 2021). Consistent with this whole-sample average, the peak of internalizing in over half (six) of the cultural groups occurred in childhood (age 8), although for one-third of the groups the peak occurred at age 17 (Rome, Naples, U.S. European American, and Thailand). There were significant cultural differences in the typicality of internalizing symptoms. Internalizing was higher than average at one or more timepoints during adolescence in the Philippines, Colombia, Thailand, and Rome. The highest levels of internalizing were found in Colombian 8-year-olds; yet, even among this group, the average internalizing score remained between “not at all true” and “somewhat or sometimes true,” indicating an objectively low average prevalence. Internalizing was lower than average at one or more timepoints in Kenya, Sweden, and among U.S. African American and U.S. Hispanic adolescents. Although we did not explore what might account for these differences, previous research suggests that the reasons might lie in cultural differences in parental expressions of warmth, or differences in expectations for and conflict over autonomy (e.g., Rothenberg et al., 2020).

With respect to externalizing (e.g., lying, truancy, vandalism, disobedience), the highest average score for the whole sample occurred among 14-year-olds, and, as with internalizing, represented a typical frequency of symptoms between “not true of me” and “somewhat or sometimes true of me” within the past 6 months. This finding is consistent with other data on typicality of externalizing, which suggest a low average prevalence (e.g., Gutman et al., 2017; Miech et al., 2022). Externalizing peaked at age 14 or 15 in most (eight) of the groups, although in three groups (Kenya, Jordan, and U.S. African American adolescents), it peaked prior to or in early adolescence (age 10 or 12). Once again, there were significant cultural differences in the typicality of externalizing symptoms. Externalizing was higher than average at one or more timepoints in Jordan, Colombia, Rome, the Philippines, and Thailand. The highest overall levels of externalizing were found in Jordanian 12-year-olds; yet, even among these youth, the average score remained between “not at all true” and “somewhat or sometimes true,” indicating an objectively low average prevalence. Externalizing was lower than average at one or more timepoints in Colombia, Sweden, U.S. African American adolescents, and U.S. Hispanic adolescents. Rothenberg et al. (2020) suggest that cultural differences in parenting practices related to warmth and behavioral control, as well as expectations and norms for risk-taking and aggression, are predictors of such cultural differences in externalizing.

In contrast to internalizing and externalizing, wellbeing was assessed only from age 12 to 17 years of age. Across cultural groups, the average score for wellbeing (e.g., engagement, perseverance, optimism) was lowest at age 15, yet, at the nadir represented a typical level of wellbeing above the midpoint of the scale, indicating that adolescents reported wellbeing as somewhat more true than untrue of themselves. This is consistent with other data on typicality of wellbeing, which suggest that wellbeing is more common than lack thereof (e.g., Van der Graaff et al., 2014; Miklikowska et al., 2022). Wellbeing hit bottom among 15-year-olds in most (nine) of the groups, although in two groups (Jordan and U.S. European American adolescents), it hit bottom at age 17. The lowest overall levels of wellbeing were found in Jordanian 17-year-olds; yet, even among these youth, the average score showed indices of wellbeing that were close to “somewhat true of me,” indicating an objectively moderate level of wellbeing. There were significant cultural differences in typicality of wellbeing. Wellbeing was higher than average at one or more timepoints in Kenya, Philippines, Sweden, and among U.S. African American adolescents. Wellbeing was lower than average at one or more timepoints in Rome, Colombia, Thailand, and among U.S. European American adolescents.

Patterns of group differences in wellbeing sometimes paralleled patterns of group differences in internalizing and externalizing, but not always. Better than average functioning across all three indices occurred in youth from Sweden and among U.S. African Americans, and Kenyan adolescents reported better than average wellbeing and internalizing, with average levels of externalizing. Lower than average functioning across all three indices occurred in youth from Colombia, Thailand, and Rome. Filipino youth reported higher than average wellbeing despite also reporting higher than average internalizing and externalizing. U.S. European American adolescents reported lower than average wellbeing despite being average with respect to internalizing and externalizing. These different patterns point to a complexity in typical adolescent behavior that merits deeper exploration to understand the cultural and psychological influences at work. For example, high levels of subjective wellbeing in the face of difficult circumstances and higher than average negative behavior might reflect adaptive preferences, or adjustment of one’s expectations based on actual constraints, not taking into account possibilities for better circumstances (Begon, 2014). In general, however, and consistent with previous research, typical functioning on all indices for adolescents was in the positive range (e.g., Gutman et al., 2017; Daly, 2021; Miech et al., 2022; Miklikowska et al., 2022). Thus, on average, a storm and stress characterization based on typicality of problem behavior and low wellbeing seems inaccurate.

### Age trajectories of internalizing, externalizing, and wellbeing across cultural groups

A storm and stress characterization of adolescents is also predicated on increases in internalizing and externalizing, and implies decreases in wellbeing, from childhood to adolescence or over the adolescent years (Arnett, 1999; Buchanan and Bruton, 2016). Although we were not able to model developmental trajectories for all the groups we studied, our findings for groups that could be modeled provide mixed support for this expectation.

The developmental trajectory for internalizing could be modeled for four groups (the Philippines, Thailand, U.S. Hispanic adolescents, and U.S. African American adolescents). There was no solid evidence for increases in internalizing from age 8–17 years in any of these countries. The internalizing trajectory from eight to 17 years of age was flat for the Philippines and for Thailand (in the latter case, although a quadratic model fit best, neither slope was significantly different from zero, essentially indicating no change over time). For Hispanic and African American adolescents from the U.S., internalizing actually declined from age 8 into early adolescence (age 10 for African American and age 12 for Hispanic youth). Thereafter, internalizing continued to decrease for African American boys, and plateaued for African American girls. In the one trajectory supporting a storm and stress characterization, internalizing among U.S. Hispanic youth increased from age 12–17, although even with the increase, internalizing never again reached the level it had been at age eight.

The developmental trajectory of externalizing could be modeled for three groups (Jordan, Philippines, and U.S. Hispanic adolescents). In the Philippines, externalizing increased from age eight to a peak at age 14, followed by slight declines. A similar pattern emerged for boys in Jordan, although the peak was at age 12. Among U.S. Hispanic youth, the level of externalizing was essentially flat; it rose slightly but not significantly from 8 to 12, and then declined significantly for girls and increased significantly for boys from 12 to 17 years. Thus, some evidence supporting a storm and stress characterization occurs in this domain, although the pattern is limited to boys in Jordan (who peak very early in adolescence) and in the U.S. Hispanic sample (whose externalizing increases beginning at age 12 through age 17 years).

The developmental trajectory of wellbeing could be tested for six groups (Jordan, Philippines, Sweden, and African American, European American, and Hispanic youth in the U.S.). In three of these groups (the Philippines, Jordan, and U.S. Hispanic) there was no significant change in wellbeing, despite what appear to be downward slopes (see Figure 3). Among U.S. African American and U.S. European American youth, wellbeing declined significantly over adolescence, although for U.S. European American youth the decline was limited to those whose fathers had moderate or high father education; there was no decline for those whose fathers had low levels of education. The developmental trajectory of wellbeing for youth in Sweden was also characterized by a significant decline from 8 to 15 years, followed by an increase from 15 to 17 years. Overall, a downward trajectory of wellbeing does seem fairly common, although not universal, among the groups we examined, which provides some support for a storm and stress characterization in some contexts. The importance of considering the context of typicality in these age changes is apparent here, however, because focusing only on the decline can be misleading. Swedish and U.S. African American youth had wellbeing scores significantly above the sample mean at age eight, and except for age 15 wellbeing in Sweden (when wellbeing dips slightly below the mean), the wellbeing of youth in both groups remains above the mean at all timepoints. U.S. European American youth, in contrast, reported wellbeing equivalent to the sample mean at age 12 years, and with the ensuing decline, had wellbeing significantly below the sample mean by age 17.

### Implications for accurately characterizing adolescence

Our findings are consistent with a storm and stress characterization of adolescence in a few ways, but inconsistent with such a characterization in several others. Consistent with our hypotheses, the most support for a storm and stress characterization occurred with respect to the developmental *trajectories* of behavior and characteristics from childhood to adolescence or across the adolescent years, and this evidence was strongest with respect to externalizing and wellbeing. Taking into account all groups, including those for whom trajectories could not be modeled, internalizing peaked at age eight in most groups, but at age 17 in four; externalizing peaked at 14 or 15 in most countries; and wellbeing was lowest at age 15 or 17 in all countries. Among those groups for whom we could model developmental trajectories, there were increases in internalizing in only one group; increases in externalizing in eight groups, most often peaking around age 14, and declines in wellbeing in three groups to a nadir by age 15 or 17 years.

This evidence consistent with a storm and stress characterization must be tempered, however, by findings concerning typicality and cultural variability. For example, the overall prevalence of internalizing and externalizing problems, on average, was low. In contrast, the prevalence of wellbeing was on the high end of the scale, on average. Thus, internalizing and externalizing were not normative in any context, whereas wellbeing was normative. Of course, there were deviations from the average. Some adolescents had more extreme scores, experiencing high or very high levels of internalizing or externalizing, or low or very low levels of wellbeing. And there were significant site differences in typicality. Nonetheless, overall, the adolescents in this sample, who represent adolescents from 11 groups across eight countries, were doing (on average) well. Furthermore, developmental trajectories of internalizing, externalizing, and wellbeing were quite variable, primarily based on cultural group but also, in a few instances, based on child gender or socioeconomic status (as measured by father education). Rothenberg et al. (2020) established cultural variability in developmental trajectories of internalizing and externalizing in this same sample from 8 to 14 years of age, and this cultural variability holds true over two more waves of data extending into late adolescence. Thus, both typicality and developmental trajectories of internalizing, externalizing, and wellbeing suggest the importance of contextual factors in development, consistent with a bioecological model of development (Bronfenbrenner and Morris, 2006). These contextual factors would seem to demand as much focus in an accurate characterization of adolescence as do the universal maturational forces predicated by a storm and stress characterization (see also Romer et al., 2017; Abrams, 2022, for similar arguments emerging in recent research on the adolescent brain).

It is notable that with respect to the age trajectories we were able to test, the best matches to a storm and stress characterization of adolescence occur among youth with western European ancestry, as we had hypothesized. These are the groups upon which storm and stress theory was initially developed (Hall, 1904) and the groups who have been most widely studied over the past century (Thalmayer et al., 2021). Specifically, the declines in self-reported wellbeing among U.S. European American and Swedish adolescents are consistent with the idea that the transitions and developmental challenges of adolescence result in decreasing positive qualities such as optimism, perseverance, and happiness. Although we could not test the growth curve for adolescents from Rome, levels of internalizing and externalizing among these youth were higher than average by mid-to late-adolescence (but not earlier). Internalizing peaked at age 17 in Naples, as well, even though this level of internalizing was not significantly different from the sample average.

In a similar vein, the differences in typicality of difficulties vs. wellbeing between U.S. European American, on the one hand, and U.S. African American and Hispanic youth, on the other, are striking. When their scores diverged from the average of the whole sample, U.S. minority adolescents had significantly *lower* levels of internalizing and externalizing, and significantly *higher* wellbeing. In contrast, U.S. European American adolescents were consistently at the sample average for internalizing and externalizing, and by age 17 their wellbeing was lower than average. These differences are especially interesting in light of the fact that U.S. minority adolescents are more likely to experience difficult life contexts (e.g., low income, racism, discrimination), and less privilege, than U.S. European American adolescents (e.g., Pachter et al., 2010; Hughes et al., 2016; Bialik and Cilluffo, 2017; The Annie E. Casey Foundation, 2021).

There are exceptions to these indications of Western youth being more susceptible to storm and stress: typicality of externalizing was lower among Swedish youth than the sample average from ages 9–14 years. And U.S. minority youth exhibited declines in wellbeing (African American adolescents) and increases in internalizing (Hispanic boys and girls) and externalizing (boys only) from 12 to 17 years. However, these changes for U.S. minority youth occurred within a context of lower overall typicality of internalizing and externalizing and higher typicality of wellbeing.

The group variability in typicality and trajectories of adolescent behavior and characteristics, both in the current study and prior literature, suggests the importance of looking toward cultural and societal predictors of storm and stress and of wellbeing. The differences highlighted above between adolescents of Western European heritage and others might have something to do with different cultural exposure to the negative stereotypes of and expectations for adolescents that emanated from storm and stress theory (Brown et al., 2002; Buchanan et al., 2013; Qu et al., 2016). Although this would be intriguing to study, clearly there are other, complex, contextual forces as well, because other comparisons illuminate higher than average problems in some non-Western youth. For example, internalizing and externalizing were significantly elevated across the adolescent years, through age 17, in Filipino youth. At some point in adolescence, internalizing and externalizing (or both) are higher than average in Colombia, Thailand, and Jordan (in addition to Rome). Youth from Colombia, Jordan, and Thailand, along with Rome and U.S. European American youth, also have lower wellbeing than average at some point in adolescence.

Altogether, the characterization of adolescence based only on trajectories of difficult behavior falls short. The data presented here point to the importance of considering both trajectory and typicality in interpretations of adolescent behavior, and of integrating a variety of contextual factors more squarely into the characterization of adolescents on which researchers, practitioners, and laypersons rely. Our own and other data suggest that these contextual factors range from cultural values (e.g., Dasen, 2000; Duell et al., 2016; Rothenberg et al., 2020) to parenting (e.g., Rothenberg et al., 2020) to personality (e.g., Branje et al., 2010; Racz et al., 2017), to community and societal pressures (e.g., Luthar et al., 2020). Although universal biological characteristics emphasized by a storm and stress characterization might play a role for some individuals (Buchanan et al., 1992; Steinberg, 2008; Casey et al., 2010), they are not deterministic, and there is a great deal of variation in levels and trajectories of both difficult and positive behavior at adolescence (see also Hollenstein and Lougheed, 2013). To the extent that biological changes and characteristics of adolescence are important in behavioral and emotional changes, their impact clearly depends on these various contextual and individual characteristics (e.g., Hollenstein and Lougheed, 2013; Romer et al., 2017).

Our data support arguments that a storm and stress characterization of adolescence should be replaced by a characterization that is more positive and nuanced (e.g., Hollenstein and Lougheed, 2013; Gutman et al., 2017). Such arguments are not new (e.g., Offer and Schonert-Reichl, 1992) and such a perspective is arguably implicit in modern theory and research on adolescence, given the influence of bioecological theories (Gutman et al., 2017; Luthar et al., 2020) as well as abundant studies documenting specific environmental influences on a variety of adolescent characteristics. It is also arguably implicit among professionals who work with adolescents and dispense advice about them, considering that many websites, podcasts, and blogs work to dispel “myths” of adolescence and offer guidance to parents about how to help their adolescents avoid problems—or even thrive. Nonetheless, a storm and stress characterization is often the starting point for descriptions of adolescence, whether in the media or in research (Buchanan and Bruton, 2016; Jewell et al., 2019). Given the danger of storm and stress stereotypes, like other stereotypes, to influence adolescents’ behavior and relationships with parents and other adults, and to produce self-fulfilling prophecies (Madon et al., 2003, 2011; Buchanan and Hughes, 2009; Qu et al., 2018; Silva et al., 2021), a concerted effort to replace storm and stress as the starting point for understanding adolescence seems imperative (see also Kendall-Taylor and Fuligni, 2022). Efforts that have been undertaken to address ageism faced by older adults (e.g., The World Health Organization, 2022) may provide a template for doing so.

One reasonable concern that has been expressed about a more positive characterization of adolescence is that normal adolescent difficulties–risk-taking, mood swings, conflict with parents—might become pathologized (Arnett, 1999). Alternatively, however, expecting difficulty to be normal might cause parents and professionals to minimize the seriousness of behavior that is, indeed, unhealthy, if not pathological. Such views might lead to delayed intervention, whether that intervention is simply a sympathetic and understanding response to emotional distress or obtaining professional help (Buchanan and Bruton, 2016), perhaps in part because of lower parenting self-efficacy among parents who have negative expectations (Glatz and Buchanan, 2015). A more positive and nuanced characterization of adolescence is arguably more accurate and more likely to promote positive development.

### Strengths, limitations, and future research

This study is unique in the extent of data over the adolescent years and with respect to sample diversity, with participants from 11 groups across eight countries. Thus, the study can address questions of the prevalence, age trajectories, and cultural diversity of both negative and positive characteristics. Nonetheless, these data have their limits. Although the samples were designed to be representative of the cities from which they were drawn, they are not nationally representative, so findings may not generalize to entire countries included in this study. We had data only from ages 8–17 for internalizing and externalizing and 12–17 for wellbeing. We cannot speak to how the trajectories would be different if they extended earlier or later in development. Within the age range and for the outcomes we were able to examine, linear change was most common, although occasionally quadratic or piecewise models suggested important transition points. These points varied by outcome and cultural group, but most commonly pointed to age 10 or 12, and thus the transition from childhood into adolescence, as a point of change, change that continued linearly until age 17. Given our aim to study typicality and trajectories between childhood and adolescence, broadly speaking, so as to address a storm and stress characterization, we have focused on the larger developmental trends. It might be valuable for future research to take a closer look at phase transitions with respect to group variability in physical, cognitive, or social changes.

The small sample size in each cultural group and small degrees of freedom in latent curve models may have led us to incorrectly reject a number of latent curve models within countries (Chen et al., 2008; Kenny et al., 2015). However, given our goal to understand heterogeneity in internalizing and externalizing and adolescent wellbeing in different cultural groups, it was important to run the models by group, not as a whole controlling for cultural group. Therefore, due to the fact that no functional forms for some sites achieved appropriate levels of model fit and also that some sites had different functional forms, we were not able to estimate trajectories for some countries and we were able to compare slopes only in countries with the same functional form.

Our analyses do not address whether different adolescents are represented among those who exhibit objectively high internalizing or externalizing, or objectively low levels of wellbeing, at some point during adolescence. Other research suggests a high degree of stability in difficulty or wellbeing across childhood and adolescence (e.g., Racz et al., 2017). Nonetheless, if most or all adolescents report objectively high levels of problems or objectively low levels of wellbeing at some point during adolescence, then a storm and stress characterization might be more appropriate. We also have not tested all predictions of a storm and stress characterization: whether difficulties and challenges decline, and wellbeing increases, from adolescence into adulthood, and whether difficulties/wellbeing during adolescence predicts better or worse functioning in adulthood. Future longitudinal research can examine these questions. It is also important to examine more closely when increases in internalizing or externalizing, or decreases in wellbeing, from what is typical for an individual child–even if not extreme in an objective sense–indicate behavior worthy of intervention.

Another potential limitation of this study is that our measures were based only on adolescents’ self-reports. Adolescents’ own sense of their wellbeing is a critical aspect of their development at this time, and of whether they experience adolescence as a time of storm and stress. Nonetheless, there are individual and country-level differences in subjective wellbeing, related to factors ranging from experiences of poverty and war to values placed on individualism or optimism (Diener et al., 1995; Standish and Witters, 2014). As noted earlier, self-reports of wellbeing can be influenced by adaptive preferences in ways that overstate actual wellbeing. External indicators of wellbeing, or more objective indicators of positive character traits should be examined in future research in order to enhance understanding of positive developments during adolescence. Furthermore, concepts of wellbeing, as well as internalizing and externalizing behaviors, are situated in cultural contexts, and typicality and trajectories of these behaviors might have different meanings in different contexts even when they appear similar. For example, in Thailand, where externalizing behaviors are more disruptive to notions of group harmony and there are strong cultural sanctions against externalizing behavior, children’s externalizing behaviors are regarded as being more problematic than in the USA, where externalizing behaviors are not regarded as being quite as problematic (Weisz et al., 2006).

In sum, we examined typicality at several points spanning late childhood to late adolescence across a large cross-cultural sample, and conclude that average levels of internalizing, externalizing, and wellbeing do not indicate adolescent storm and stress on average. Despite some developmental trajectories that were consistent with a storm and stress characterization, typical behavior in all groups across adolescence was more positive than negative. Furthermore, many developmental trajectories did not indicate increasing problems. After more than a century dominated by a storm and stress characterization that emanated from and was perpetuated by Western European theorists and researchers, it is time to take a more positive and nuanced characterization of adolescence seriously.

## Data availability statement

The data analyzed in this study are subject to the following licenses/restrictions: Approval from Parenting Across Cultures research team. Requests to access these datasets should be directed to AS, askinner@duke.edu.

## Ethics statement

This study was approved by the Duke University Institutional Review Board and Ethics Committees at universities in all countries in which data were collected. Written informed consent to participate in this study was provided by the participants’ legal guardian/next of kin.

## Author contributions

CB conceptualized the manuscript and drafted the manuscript. SZ performed statistical analyses and contributed to the writing of the manuscript. JL and AS contributed to the writing of the manuscript. CP, DB, ES, KD, KD-D, LD, LS, LC, LA, LU, MB, PO, QLo, QLi, SY, SG, ST, and SA-H participated in the design of the study and supervised data collection. All authors read and approved the final manuscript.

## Footnotes

1 In what follows, cultural differences for internalizing and externalizing that occurred from 10 to 14 years of age are the same as those reported by Rothenberg et al. (2020), but additional differences were uncovered in the 15 and 17-year-old data; we report all differences here, for ease of summary.
